# JCAD expression and localization in human blood endothelial cells

**DOI:** 10.1016/j.heliyon.2020.e05121

**Published:** 2020-10-08

**Authors:** Manabu Shigeoka, Satomi Arimoto, Masaya Akashi

**Affiliations:** a Department of Oral and Maxillofacial Surgery, Kobe University Graduate School of Medicine, Kobe, Japan; b Division of Pathology, Kobe University Graduate School of Medicine, Kobe, Japan

**Keywords:** JCAD (junctional cadherin 5 associated), Blood endothelial cell-cell junction, Inflammation, Submandibular gland, Cardiology, Cardiovascular system, Pathology, Oral medicine

## Abstract

**Background:**

Junctional Cadherin 5 Associated (JCAD) is an endothelial, cell-cell junction protein, and its expression is associated with cardiovascular diseases including atherosclerosis and hypertension. However, to date, there are few studies confirming JCAD expression and precise localization in human tissues by immunohistochemical staining.

**Methods:**

JCAD expression and localization was assessed in four human submandibular gland (SMG) specimens by immunohistochemical staining. One specimen of SMG with sialoadenitis was accompanied by severe inflammation and fibrosis, while the other was largely normal. Other two SMGs were accompanied by severe fibrosis because of irradiation.

**Results:**

Immunohistochemical analysis of human SMGs revealed JCAD localization at the blood endothelial cell-cell junctions. JCAD expression was more evident in microvessels and arteries in areas affected by inflammation.

**Conclusions:**

The localization of JCAD at endothelial cell-cell junctions was confirmed in human tissues. JCAD expression may be affected by pathological conditions.

## 1 Introduction

Junctional Cadherin 5 Associated (JCAD, previously known as Junctional protein associated with Coronary Artery Disease and KIAA1462) was first identified as a novel component of endothelial cell-cell junctions [1]. Single nucleotide polymorphisms in the human *JCAD* gene associated with coronary artery disease were reported in two large-scale genetic studies [2, 3]. The JCAD protein lacks obvious domain structures with predicted functions. Although the function of JCAD remains incompletely understood, several studies have reported possible functions. *Jcad*^*−/−*^ mice exhibit significantly smaller tumor volumes and less intratumoral neovascularization compared with wild-type mice following subcutaneous injection of mice with melanoma, lung carcinoma and breast cancer cells, indicating that JCAD may play a role in pathological angiogenic processes, but not developmental angiogenesis [4]. JCAD knockdown in human umbilical vein endothelial cells increased apoptosis and reduced proliferation, migration and angiogenesis by negatively regulating Hippo signaling [5]. In a study using *Jcad*^*−/−*^ *ApoE*^*−/−*^ mice, JCAD deficiency attenuated high-fat-diet-induced atherosclerosis in ApoE-deficient mice, and also improved acetylcholine-mediated endothelium-dependent relaxation, but not sodium nitroprusside-mediated smooth muscle cell relaxation [6]. In a separate study using *Jcad*^*−/−*^ *ApoE*^*−/−*^ mice, JCAD deficiency caused a significant reduction in atherosclerosis in the aortic arch inner curvature [7]. In JCAD knockout mice, there was a decrease in expression of proinflammatory adhesion molecules such as Vcam-1 and Icam at sites of disturbed flow on the endothelial cell layer and recovery after hind limb ischemia [7]. The acute response to shear stress was also reduced following JCAD knockdown in primary human, aortic endothelial cells [7]. Taken together, higher JCAD expression may lead to increased expression of pro-proliferative, anti-apoptotic and pro-inflammatory genes, and promote endothelial dysfunction and atherogenesis [5].

Several studies have also demonstrated roles for JCAD in tumorigenesis and Alzheimer's disease. One study reported high expression of JCAD in multiple hepatoma cell lines and in pre-carcinoma lesions in a mouse model of non-alcoholic steatohepatitis (NASH), as well as in human NASH-associated hepatocellular carcinoma, in contrast to the low levels of expression in normal human liver [8]. Another study reported that *JCAD* may play a pathogenic role in ovarian serous borderline tumors, an intermediate stage between benign cystadenoma and invasive low-grade serous carcinoma [9]. *JCAD* was also reported to be a candidate gene involved in late-onset Alzheimer's disease in APOE carriers [10, 11], although differential expression of JCAD was not observed in brain tissue from these patients [11].

Importantly, most studies involving JCAD have not shown the expression and precise localization of JCAD in human tissues with immunohistochemical staining, with the exception of one study demonstrating intimal endothelial JCAD expression in advanced human atherosclerosis plaques [6]. We hypothesized that JCAD expression may be elevated in blood endothelial cells in pathological versus normal conditions. In this study, we examined the expression of JCAD in surgical specimens of submandibular glands (SMGs) accompanied by inflammation or fibrosis by immunohistochemical staining.

## 2 Methods

Four excised, human SMG samples were selected for the current study. Patient details are shown in Table 1. SMG in Case 1 was accompanied by severe inflammation and fibrosis, while SMG in Case 2 was accompanied by moderate inflammation. Both Case 3 and 4 received radiation therapy for oropharyngeal carcinoma. Their SMGs were accompanied with severe fibrosis. All four patients had no vascular diseases such as hypertension. This study (No. 160141) was approved by the Medical Ethics Committee of Kobe University Hospital. All subjects provided written informed consent for release of clinical information and bone samples for this study.

**Table 1:** Characteristics of pathological submandibular glands included in this study.

Case	Sex	Age	Primary disease	Histopathological characteristics	
				Fibrosis	Inflammation
1	M	27	Sialoadenitis caused by sialolithiasis	++	++
2	F	50	Mandibular defect after osteomyelitis surgery∗	-	+
3	M	70	Mandibular osteoradionecrosis∗	++	+
4	M	81	Mandibular osteoradionecrosis∗	++	+

∗ Submandibular glands were removed from this patient for identification of the recipient vessels for free fibula flap transfers.

All SMG specimens were 10% formalin-fixed without freezing for 24–48 h after surgery, and serial sections with a thickness of 3–4 μm were prepared. Histological factors including fibrosis and inflammation were assessed in hematoxylin and eosin-stained sections by two authors (M.S. and S.A.), including one pathologist blinded to clinicopathological data. Briefly, representative areas with notable changes related to each factor were identified by scanning the SMG sections at low magnification. Grading was then performed on a 200× field using the following system: severe (++); moderate (+); weak or none (-).

Immunohistochemical analysis of JCAD expression in SMG specimens was assessed using the EnVision Dual Link System-HRP, 3,3-diaminobenzidine (Dako, Glostrup, Denmark). JCAD antibody staining conditions were optimized prior to immunohistochemical analysis of SMG sections using previously published antibodies: anti-human rabbit polyclonal antibody (HPA017956^5,7^; Sigma-Aldrich, St. Loius, MO); anti-human rabbit polyclonal antibody (ab121545^4,8^; Abcam, Cambridge, MA). Antigen activation of all specimens was performed by heat treatment at 95 °C or 121 °C using Dako Target Retrieval Solution (pH 9, 10×; S2367; Dako, Glostrup, Denmark) or CB buffer adjusted its pH of Citric Acid Monohydrate (091-06; Nacalai Tesque, Kyoto, Japan) to 6.0 with NaOH, and optimal conditions were selected by authors M. S. and M.A. Immunohistochemical staining of CD31 (150×; M0823; Dako, Glostrup, Denmark) was also performed in the same specimens. For double immunofluorescence staining of JCAD and CD31, anti-human JCAD rabbit polyclonal antibody (5×; HPA017956) and anti-human CD31 mouse monoclonal antibody (100×; JC70A; Abcam, Cambridge, MA) were used. Secondary antibodies conjugated with Alexa 594 and 488 (500×) were purchased from Invitrogen (Waltham, MA). A laser-scanning microscope (LSM700; Carl Zeiss, Oberkochen, Germany) and the LSM software ZEN 2009 (Carl Zeiss) were used for fluoroscopic observation.

## 3 Results

**Figure 1:**
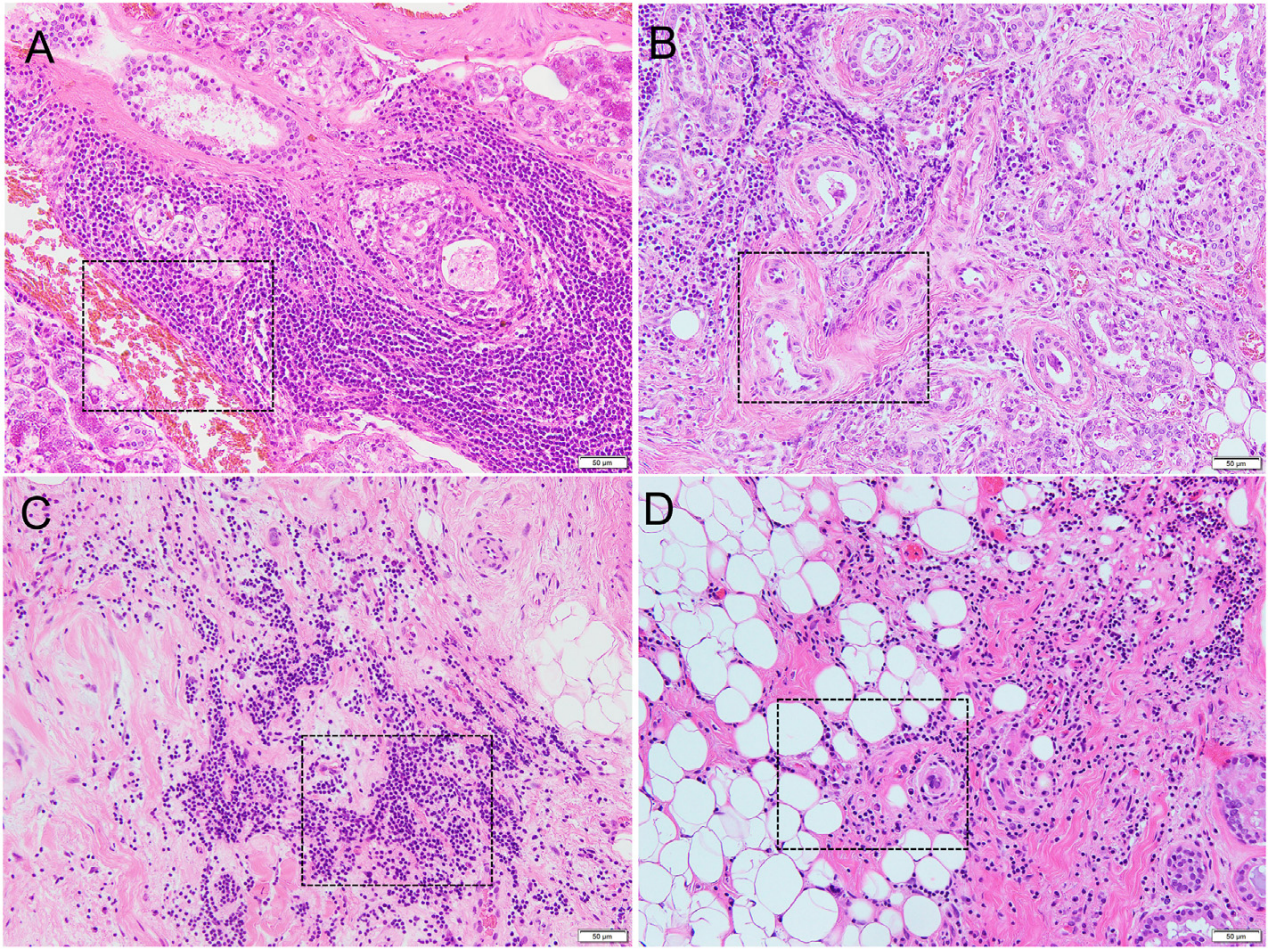
Microscopic images of submandibular gland (SMG) with hematoxylin and eosin staining. (A) Case 1. SMG is accompanied by severe inflammation and fibrosis. (B) Case 2. Predominantly normal SMG is accompanied by focal inflammation. Case 3 and 4 are irradiated SMGs, so both are accompanied by severe fibrosis (C and D). In Case 4, fatty degeneration is apparent (D). Areas indicated with a dotted black box are shown in Figures 2, 3, 4, and 5 (Scale bar: 50 μm).

In this study, we first determined the optimal conditions for immunohistochemical detection of JCAD in human SMG specimens utilizing the HPA017956 antibody with Target Retrieval Solution (pH 9, 10×) at 121 °C. The histopathological characteristics of each specimen are shown in Table 1. Microscopic analysis of SMG sections shows that Case 1 is accompanied by severe inflammation and fibrosis (Figure 1A), while the glandular tissue of Case 2 is predominantly normal, although focal inflammation was observed (Figure 1B). Both irradiated SMGs were accompanied by severe fibrosis (Figure 1C, D). In particular, fatty degeneration was found in Case 4 (Figure 1D).

**Figure 2:**
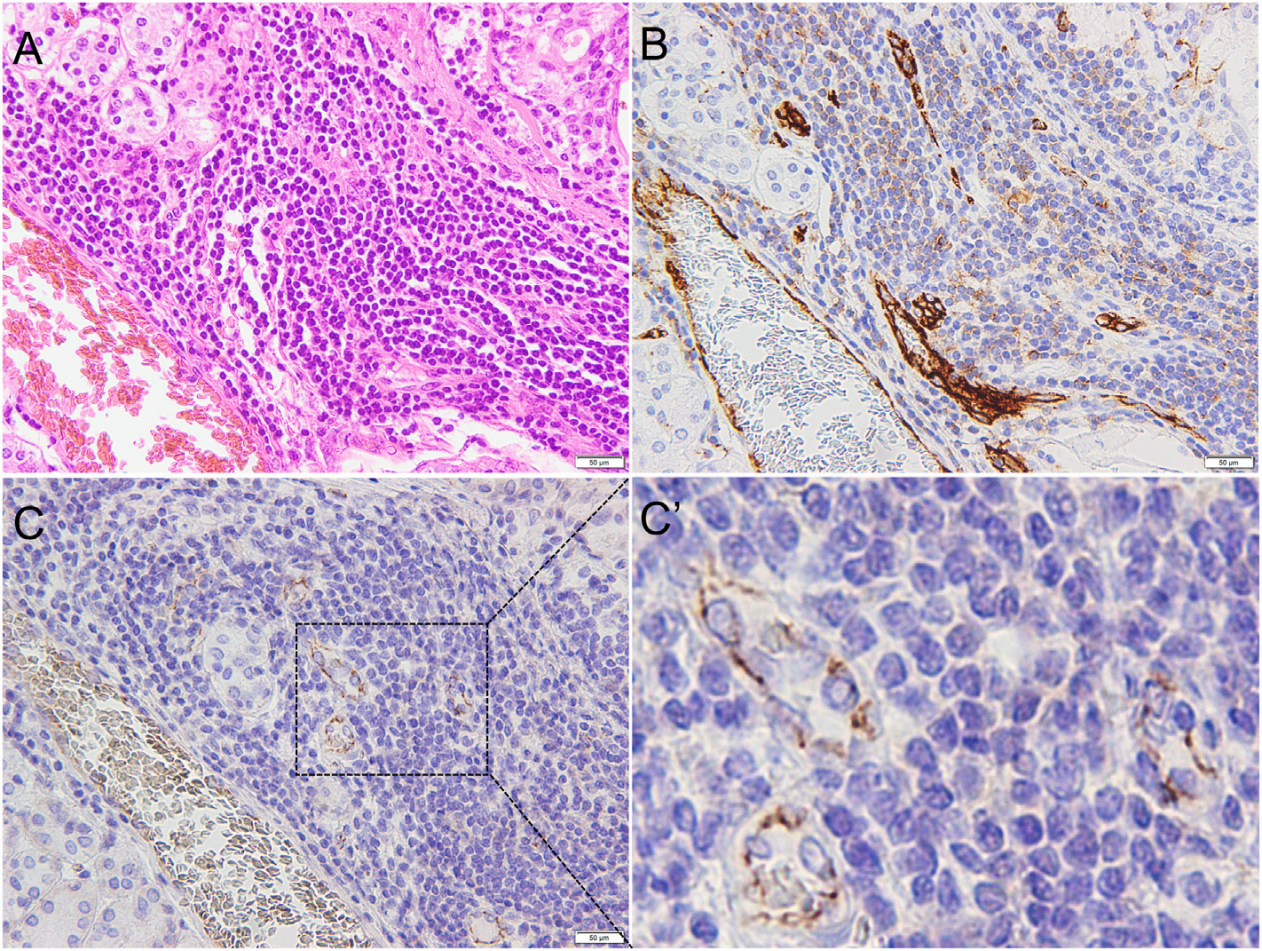
Case 1. (A) Hematoxylin and eosin staining. (B) CD31. (C) JCAD. Areas indicated with a dotted black box are shown in Figure 2C’. (C’) Enlarged view showing the expression and localization of JCAD at the endothelial cell-cell junctions of microvessels in area of inflammation (Scale bar: 50 μm).

In SMG sections from Case 1, lymphocyte infiltration was prominent around the acinar cells (Figure 2A). CD31 expression was easily detected by immunohistochemistry in the blood vessels (Figure 2B), while the JCAD signal was relatively weak when observed under low magnification (Figure 2C). However, at high magnification, JCAD expression was observed at the blood endothelial cell-cell junctions, evidenced by the presence of JCAD staining in the microvessels in areas with inflammation (Figure 2C’).

**Figure 3:**
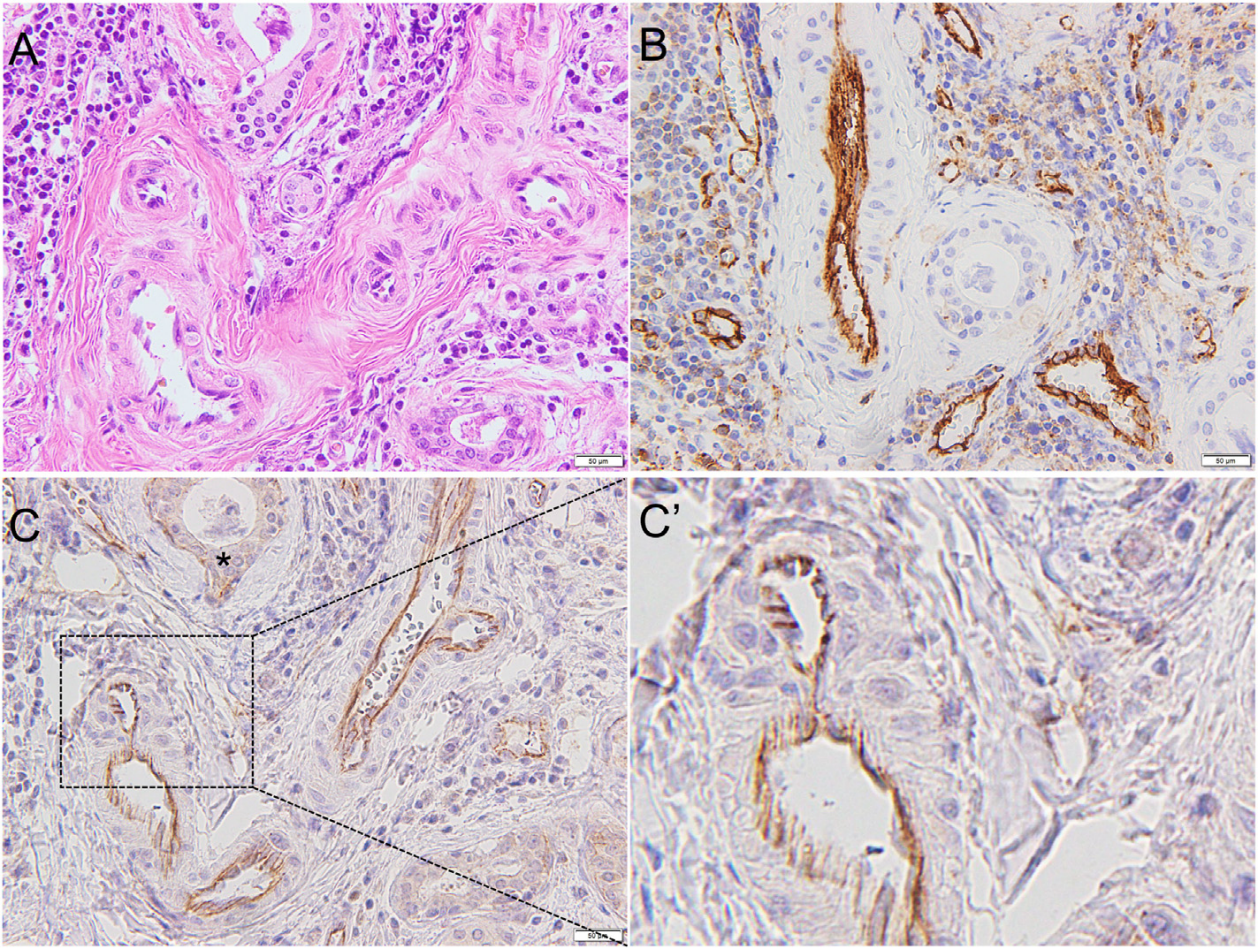
Case 2. (A) Hematoxylin and eosin staining. (B) CD31. (C) JCAD. Moderate JCAD expression is observed in the cytoplasm of ductal cells (asterisk). The areas indicated a with dotted black box are shown in Figure 3C’. (C’) Enlarged view of JCAD. JCAD is detected in the arteries in areas of inflammation. (Scale bar: 50 μm).

In SMG sections from Case 2, inflammation was moderate (Figure 3A). Immunohistochemical staining of Case 2 SMG revealed strong staining for CD31 (Figure 3B) and moderate JCAD staining at low magnification (Figure 3C) in the blood vessels. At high magnification, JCAD expression was evident at the apical side of the artery lumen in a characteristic zigzag formation (Figure 3C’).

**Figure 4:**
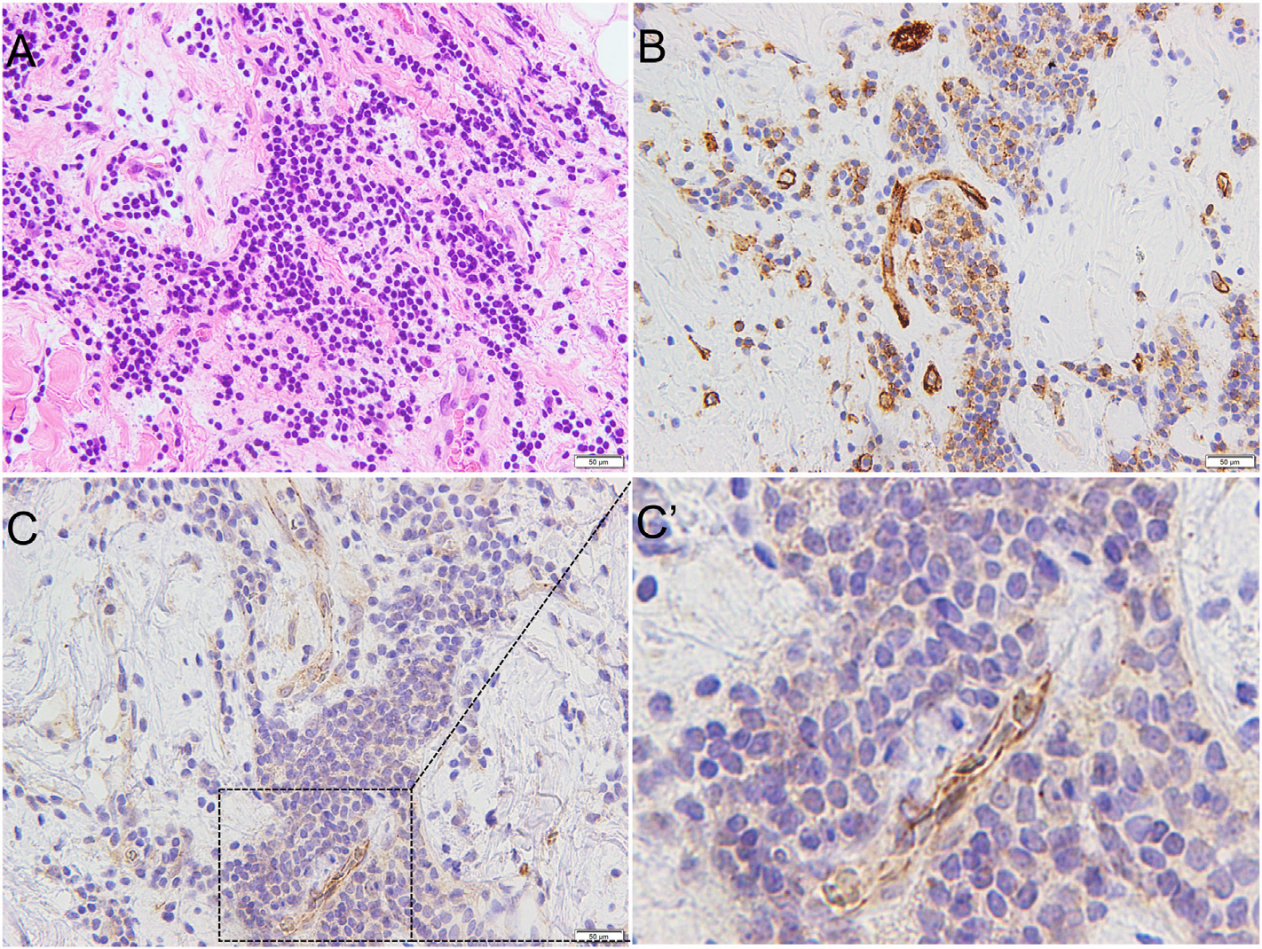
Case 3. (A) Hematoxylin and eosin staining. (B) CD31. (C) JCAD. The areas indicated a with dotted black box are shown in Figure 4C’. (C’) Enlarged view of JCAD. JCAD is detected in the microvessels in areas of inflammation. (Scale bar: 50 μm).

**Figure 5:**
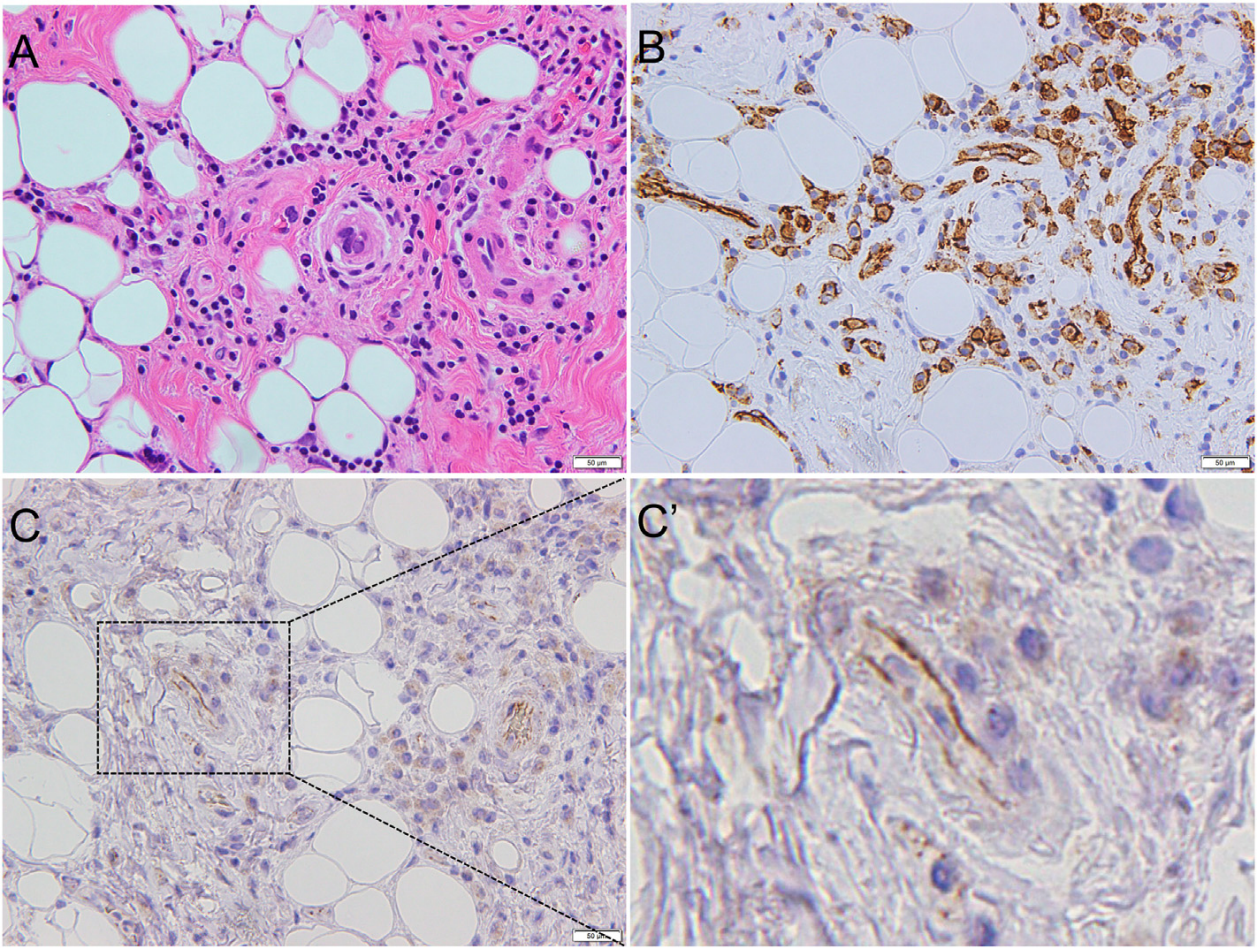
Case 4. (A) Hematoxylin and eosin staining. (B) CD31. (C) JCAD. The areas indicated a with dotted black box are shown in Figure 5C’. (C’) Enlarged view of JCAD. JCAD is detected in the microvessels in areas of inflammation. (Scale bar: 50 μm).

In SMG sections from Case 3 and 4, severe fibrosis and moderate inflammation were found (Figures 4A and 5A). Similarly, immunohistochemical staining of Case 3 and 4 SMGs revealed strong staining for CD31 in the numerous blood vessels (Figures 4B and 5B). JCAD expression was weak at low magnification (Figures 4C and 5C), but could be detected in the blood vessels in the areas with inflammation at high magnification (Figures 4C’ and 5C’).

**Figure 6:**
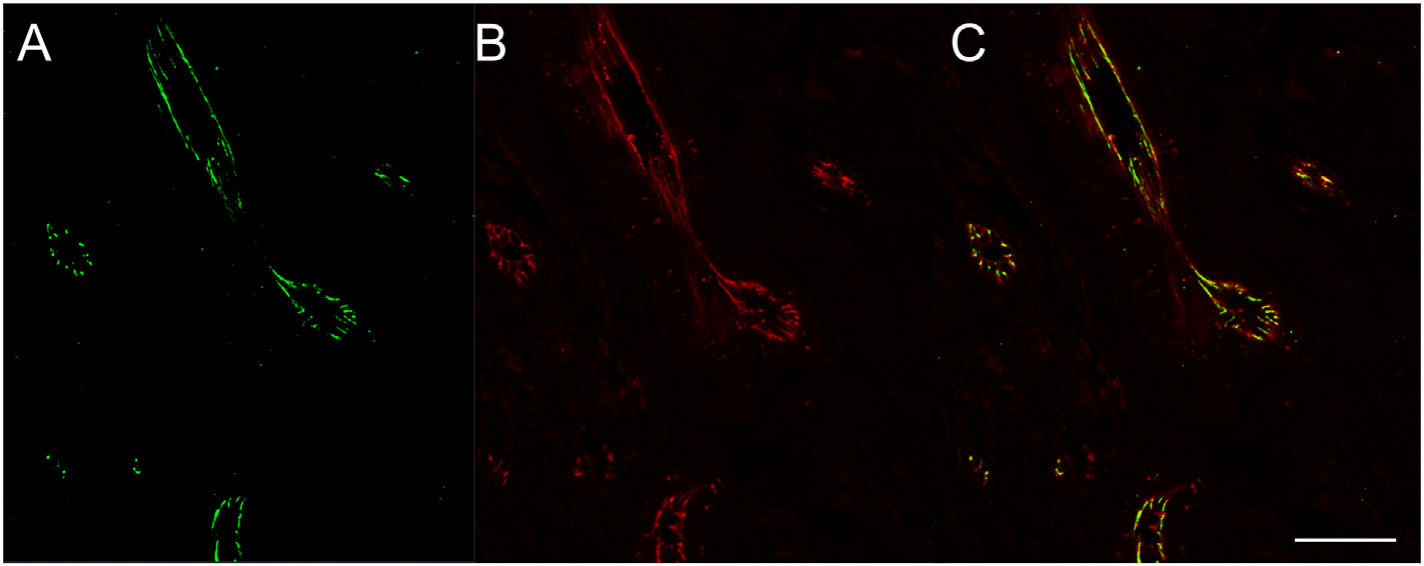
Case 1. Double immunofluorescence staining of JCAD and CD31. (A) JCAD (green) and (B) CD31 (red). (C) Merged image. (Scale bar: 50 μm).

Finally, co-localization of JCAD and CD31 at blood endothelial cell-cell junctions in Case 1 was confirmed in the double immunofluorescence staining (Figure 6).

## 4 Discussion

In addition to its role in cardiovascular disease, there is also evidence to suggest a role for JCAD in tumorigenesis [1, 2, 4, 5, 6, 7, 8, 9]. While previous studies have shown that JCAD localizes at endothelial cell-cell junctions in murine tissues and in cultured human blood endothelial cells [3, 4, 6, 7], this has not been confirmed in human tissues. To our knowledge, this is the first study confirming JCAD expression in blood endothelial cells from human tissues and its localization at cell-cell junctions.

Previous studies have shown that JCAD plays a role in angiogenesis under pathological conditions [4]; therefore, we investigated JCAD expression in the vascular cells of SMGs accompanied by inflammation or fibrosis. SMG of Case 1 was accompanied by severe inflammation and fibrosis, and that of Case 2 was almost normal. SMGs of Case 3 and 4 were irradiated, so they were accompanied by severe fibrosis. JCAD expression was detected in the arteries and the microvessels especially in areas of inflammation when examined under high magnification. Although studies have shown that JCAD is expressed in cultured human endothelial cells from the artery [7] and vein [4, 5], in humans, JCAD expression may be affected by the pathological status of blood vessels.

Similar to JCAD, several well-characterized vascular proteins are known to be regulated by surrounding conditions. Endoglin (CD105) is an accessory receptor for transforming growth factor beta, and expression is detected in actively proliferating cells including intratumoral vascular endothelial cells [12, 13]. Endoglin expression increases following hypoxia, and is thought to play an essential role in modulating angiogenesis in response to ischemia [14]. Endoglin plays a role in regulating vascular permeability, and its complete absence leads to disruption of VE-cadherin-mediated endothelial cell-cell junctions [15]. Nestin, an intermediate filament protein, is an angiogenesis-specific marker, and its expression is detected in newly formed blood vessels within tumor tissues including pancreatic cancers; however, expression is not observed in intratumoral mature vessels [16, 17]. Of interest, nestin-expressing endothelial cells have also been identified in small caliber blood vessels in the peri-infarct/infarct region of ischemically-damaged human heart tissue [18, 19]. Both endoglin and nestin knockout mice are embryonic lethal (the former dies at E11.5 with defective vascular development [20], and the latter dies at E8.5 from neural stem cell apoptosis [21]), indicating that both proteins are essential for development. In contrast, JCAD knockout mice show normal development and reproduction capacity [4].

Finally, some limitation of this study must be mentioned. In this study, we also showed that moderate expression of JCAD is only detected in the ductal cells and expression is localized to the cytoplasm (Figure 3C), but not at cell-cell junctions. In mice, JCAD is expressed in Sertoli cells in the seminiferous tubules of the testes, and in the perineurium in the sciatic nerve at the cell-cell junctions [3]. With the exception of endothelial cells, to date the function of JCAD in other tissues remains elusive. Additionally, the number of samples in this study was so small. Although the function of JCAD is not completely understood, its role, especially in circulatory diseases such as hypertension [21] and atherosclerosis [22], has attracted significant attention. JCAD induces the expression of inflammatory genes and drives an inflammatory process [23]. To reveal the potential role of JCAD as a marker of pathological blood endothelial cells, large scale immunohistochemical comparison of JCAD expression between human normal and pathological tissues should be performed.

As conclusions, we confirmed JCAD expression and localization in human tissues. Immunohistochemical staining of SMGs revealed that JCAD expression was more evident in the arteries and microvessels of tissues affected by inflammation than in areas with few inflammation, suggesting that JCAD may be a marker of pathological blood endothelial cells.

## Declarations

### Author contribution statement

Manabu Shigeoka: Performed the experiments; Analyzed and interpreted the data; Contributed reagents, materials, analysis tools or data; Wrote the paper.

Satomi Arimoto: Performed the experiments; Contributed reagents, materials, analysis tools or data.

Masaya Akashi: Conceived and designed the experiments; Performed the experiments; Analyzed and interpreted the data; Contributed reagents, materials, analysis tools or data; Wrote the paper.

### Funding statement

This work was supported by Grants-in-Aid for Scientific Research (C) 18 (Grant no. 19K10331).

### Competing interest statement

The authors declare no conflict of interest.

### Additional information

No additional information is available for this paper.
